# Reactions Between Chalcogen Donors and Dihalogens/Interalogens: Typology of Products and Their Characterization by FT-Raman Spectroscopy

**DOI:** 10.1155/BCA/2006/58937

**Published:** 2006-12-31

**Authors:** Massimiliano Arca, M. Carla Aragoni, Francesco A. Devillanova, Alessandra Garau, Francesco Isaia, Vito Lippolis, Annalisa Mancini, Gaetano Verani

**Affiliations:** Dipartimento di Chimica Inorganica ed Analitica, Università degli Studi di Cagliari, S.S. 554 Bivio per Sestu, 09042 Monserrato, (CA), Italy

## Abstract

The chemical bond and structural features for the most important classes of solid products obtained by reacting chalcogen donors with dihalogens and interhalogens are reviewed. Particular attention is paid to the information the FT-Raman spectroscopy can confidently give about each structural motif considered in the absence of X-ray structural analyses.

## INTRODUCTION

Reactions of dihalogens (I_2_, Br_2_) and interhalogens (IBr, ICl) with organic molecules containing group 16-donor atoms (LE; L = organic framework, E = S, Se) have received renewed interest in recent years. This is due to two principal reasons: their intrinsic interest and their implications in different fields of research which span from synthetic to biological, material, and industrial chemistry. For example, 1-methyl-imidazole-2-thione and related molecules show considerable antithyroid activity in vivo via I_2_ complexation [1–3]; I_2_ and IBr adducts of perhydrodiazepine-, and piperazine-2, 3-dithione derivatives can oxidize gold(0), palladium(0), and platinum(0) [4–6]; a similar activation of metal(0) powders is observed with the I_2_ adducts of phosphine sulfide compounds [7–9]; complexes between I_2_ and sulfur containing molecules can have interesting electrical properties [10].

**Figure 1:**
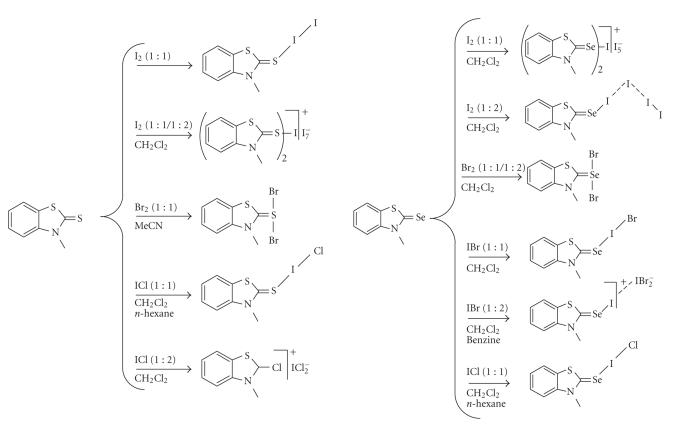
Schematic representation of the compounds obtained from the reactions of *N*-methylbenzothiazole-2(3H)-thione (**1**) and *N*-methylbenzothiazole-2(3H)-selone (**2**) with I_2_, Br_2_, IBr, or ICl characterized by X-ray diffraction analysis. This scheme must be intended purely as an illustration of the various compounds with no implications on the nature of the chemical bonds involved.

These reactions, particularly using chalcogenone donors featuring a >C=E (E = S, Se) double bond can follow a variety of pathways depending on both the acid/base nature of the reactants and the experimental conditions used, the most important one being the formation of neutral charge-transfer (CT) “spoke” adducts featuring almost linear E−X−Y moieties [X = Y = I, Br or X = I, Y = Br, Cl (E = S); X = Y = I or X = I, Y = Br, Cl (E = Se)] [5, 6, 11–84] or insertion adducts containing “T-shaped” X−E−Y fragments [X = Y = Br, Cl (E = S); X = Y = I, Br, Cl, or X = I, Y = Br (E = Se); other X−E−Y hypervalent compounds are obtained by different synthetic strategies, see Devillanova et al in this issue of BC&A] [59, 65, 66, 69, 71, 85–89]. Other different structural archetypes have also been established by X-ray diffraction analysis for the products of these reactions; they mainly include ionic products such as two-chalcogen-coordinated halogen(I) complexes [(LE−X−EL)^+^] [43, 45, 65, 90], and dications containing a chalcogen-chalcogen single bond [(LE−EL)^2+^] [3, 65, 72, 91, 92]. Polyhalides of exciting structural complexity can be found as counteranions of these ionic compounds [3, 91, 92]. A significant example is represented by the distribution of products from the reactions of *N*-methylbenzothiazole-2(3H)-thione (**1**) and *N*-methylbenzothiazole-2(3H)-selone (**2**) with I_2_, Br_2_, IBr, or ICl (Figure 1).

This variety of products, besides being very puzzling from a kinetic and thermodynamic point of view [66, 77, 88, 89, 93], represents a serious challenge when it comes to characterize the outcome of the reactions between chalcogen-donor ligands and dihalogens and interhalogens, especially when an X-ray crystal structure determination is not possible. The FT-Raman spectroscopy was proved to be of particular help in giving qualitative structural information particularly in the case of compounds from reactions with diiodine [65]. However, a confident correlation between structural features and vibrational properties requires the analysis of a large number of crystallographically characterized compounds for each structural motif available.

Here we will not attempt to give an overview of all of the knowledge on the reactivity of chalcogen-donor molecules towards dihalogens and interhalogens; instead, we will focus our attention exclusively on the chemical bond and structural features, and on the main information the FT-Raman spectroscopy can confidently give about each particular structural motif so far characterized for the compounds obtained by reacting chalcogen donors with dihalogens/interhalogens.

## DISCUSSION

### Charge-Transfer adducts

Most of the reported structurally characterized neutral CT adducts have sulfur as the donor atom and diiodine as the acceptor molecule [5, 11–61]. Those obtained from molecules containing selenium and diiodine are less numerous [14, 25, 69–81], while few adducts of S- and Se-donors with IBr [57–60, 62–67, 81–83] and ICl [60, 61, 67, 68, 83, 84] have been reported and structurally characterized in the literature. Three Br_2_ adducts of S-donors have been characterized by X-ray diffraction analysis [65, 94], and no CT adducts of Te-donors are known with any dihalogen or interhalogen.

The interaction between LE chalcogen-donor molecules (E = S, Se) and XY dihalogens and interhalogens (X = Y = I, Br; X = I, Y = Br, Cl) to give adducts containing an almost linear E−X−Y fragment can be seen as a charge-transfer process. It occurs via the transfer of charge density from a lone pair of electrons on the donor atom to the empty σ* orbital of the halogen species, producing a lowering in the X−Y bond order. The consequent increase in the X−Y bond length can be finely tuned by using donors of different strengths, which means changing either the chalcogen-donor atom or its chemical environment.

**Figure 2:**
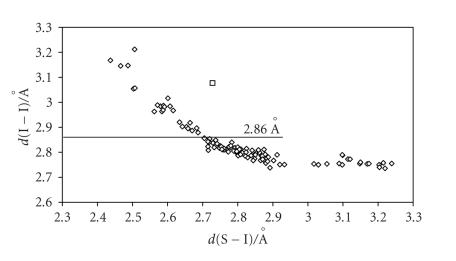
Scatter plot of *d*(I−I) versus *d*(S−I) for all structurally characterized I_2_ adducts with S-donors reported in the literature: (⋄) [5, 11–44, 46–61]; benzimidazole-2(3H)-thione ·I_2_(□) [45].

**Figure 3:**
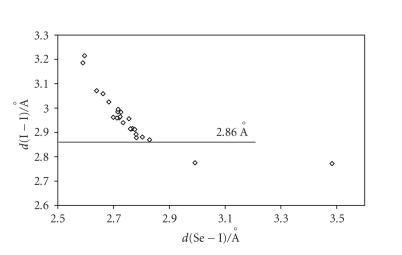
Scatter plot of *d*(I−I) versus *d*(Se−I) for all structurally characterized I_2_ adducts with Se-donors reported in the literature [14, 25, 69–81].

**Figure 4:**
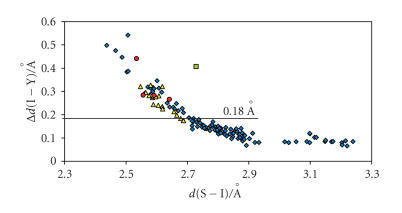
Scatter plot of Δ*d*(I−Y) [Δ*d*(I−Y) = *d*(I−Y)_adduct_ − *d*_0_ (I−Y)_gas phase_ (Y = I, Br, Cl)] versus *d*(S−I) [Y = I (⋄) [5, 11–44, 46–61], Br (Δ) [57–60, 62–67, 81–83], Cl(o) [60, 61, 67, 68, 83, 84]; *d*_0_ (I−I)_gas phase_ = 2.67 Å [95], *d*_0_ (IBr)_gas phase_ = 2.47 Å [95], *d*_0_ (ICl)_gas phase_ = 2.32 Å [95]]; benzimidazole-2(3H)-thione ·I_2_ (□) [45].

**Figure 5:**
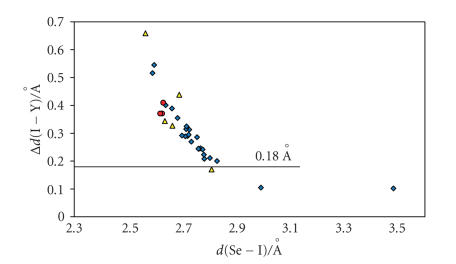
Scatter plot of Δ*d*(I−Y) [Δ*d*(I−Y) = *d*(I−Y)_adduct_ − *d*_0_(I−Y)_gas phase_ (Y = I, Br, Cl)] [95] versus *d*(Se−I) [Y = I(⋄) [14, 25, 69–81], Br(Δ) [81–83], Cl(o) [68, 83, 84]].

Under such circumstances, the E−X and X−Y bond distances should be strongly correlated in CT adducts. In fact, a scatter plot of *d*(S−I) against *d*(I−I) distances (Figure 2) for all I_2_ adducts with sulfur-containing molecules (including those featuring I_2_ bridging two donor molecules, and those featuring chains of I_2_ molecules anchored to a donor molecule) shows a close relationship between these two distances [63, 65, 68], which initially was defined as a hyperbola-like [24]. A similar relationship is found between *d*(Se−I) and *d*(I−I) for all I_2_ adducts with Se-donors (Figure 3) [65, 68]. Analogous relationships should be expected for IBr and ICl adducts with S- and Se-donors, but the number of the reported structures is so low that it is not possible yet to establish them conclusively. However, it is possible to demonstrate that the structural features of the E−I−Y moiety (E = S, Se, Y = I, Br, Cl) for I_2_, IBr, and ICl adducts are subject to the same kind of relationship, by considering the net increase in the I−Y bond distances upon coordination Δ*d*(I−Y) instead of the absolute *d*(I−Y) value [Δ*d*(I−Y) = *d*(I−Y)_adduct_ − *d*(I−Y)_gas phase_] [63, 65–68]. In fact, the scatter plot of Δ*d*(I−Y) versus *d*(E−I) (Figures 4 and 5) clearly indicate that for both S- and Se-donor molecules, the *d*(E−I) and *d*(I−Y) bond distances (E = S, Se; Y = I, Br, Cl) observed within CT adducts with IY acceptor molecules are correlated and show the same degree of variability.

The experimental data in Figures 4 and 5, except those for I_2_ adducts characterized by bridging I_2_ molecules (E−I distances lying between 3.01 and 3.30 Å and I−I distances between 2.74 and 2.79 Å, E = S, Se) [65] and the data for the adduct benzimidazole-2(3H)-thione·I_2_ [45], can be fitted very well to the equation [66]

(1) $$\text{Δ}d\left(\text{I}-\text{Y}\right)=-b_{1}\text{ln}\left\{1-\exp\left[\frac{\left(d_{0}\left(\text{E}-\text{I}\right)-d\left(\text{E}-\text{I}\right)\right)}{b_{2}}\right]\right\}$$

obtainable by assuming a valence (bond order) model for the description of the E−I−Y system within CT adducts, with *n*(I−Y) + *n*(E−I) = 1 (E = S, Se; *n* = bond order) [12, 77], with *d*_0_(E−I) = 2.396 Å and 2.528 Å (experimental values for E = S, and Se, resp) [12], *b*_1_ and *b*_2_ are parameters.

The exclusion of the experimental data for CT I_2_ adducts characterized by I_2_ bridging two-donor molecules from the fitting procedure is justified by the fact that these systems are very different from the usual CT adducts in terms of MO description. In fact, the consequence of extending the simple *n* → σ* description for the donor/acceptor interaction in terminal I_2_ adducts to a system in which an I_2_ molecule bridges two-donor molecules (*n* → σ* ← *n*) is that only two electrons have a bonding nature, since the other two occupy a nonbonding orbital. However, these two bonding electrons are distributed over three bonds instead of over two, and much longer S ⋯ I and shorter I−I bond distances are expected [22, 65]. On the other hand, the fact that the structural data for the adduct benzimidazole-2(3H)-thione·I_2_ do not fit the generalized Δ*d*(I−Y) versus *d*(S−I) correlation (Figures 2 and 4) can be accounted on the basis of the fact that, in this compound, the terminal iodine atom is strongly H-bonded to an adjacent and symmetry-related adduct unit [45]. This interaction lengthens both the S−I and the I−I bonds with respect to the values generally observed in terminal I_2_ adducts. In fact, in this adduct, the sum of S−I and I−I distances is 5.81 Å, which is quite different from the value of 5.61 ± 0.05 Å [66] obtained by averaging the values for the other “spoke” I_2_ adducts reported in the literature (5.34 ± 0.03 Å is the average value for the sum of S−I and I−Br in IBr adducts, 5.22 Å is the average value for the sum of S−I and I−Cl in ICl adducts, whereas 5.70 ± 0.04, 5.53, and 5.33 Å are the average values for the corresponding sums for I_2_, IBr, and ICl adducts with Se-donors, resp, standard deviation is reported only for mean values obtained by averaging a conspicuous number of data (more than 10)). Interestingly, for the adduct 5-chloro-benzimidazole-2(3H)-thione·I_2_ [44], where the terminal iodine atom also participates in a strong hydrogen bond, the sum of S−I and I−I distances is 5.55 Å, and the structural parameters very well fit the generalized Δ*d*(I−Y) versus *d*(S−I) correlation.

CT I_2_ adducts (the most numerous) were classified into three categories [34, 65]. (i) Weak or medium-weak adducts characterized by a mutual perturbation effect between the donor and the I_2_ molecules. The I−I bond order [*n*(I−I)], defined by the equation *d*(I−I) = *d*_0_ − *c* log *n* (where *d*_0_ is the I−I bond distance for I_2_ in the gas phase and *c* is an empirical constant with a value of 0.85), in these systems ranges from values slightly lower than 1 (unperturbed I_2_ molecule, *d*(I−I) = 2.715(6) Å in the solid state) [96] to no less than 0.6 (*d*(I−I) < 2.86 Å). (ii) Strong adducts characterized by *n*(I−I) ranging between 0.4 and 0.6 (2.86 Å < *d*(I−I) < 3.01 Å). (iii) Very strong adducts in which the donor-acceptor interaction is so strong that *n*(I−I) becomes lower than 0.4 (*d*(I−I) > 3.01 Å). Figures 2 and 3 clearly show that I_2_ adducts with S-donors are mainly weak adducts, whereas those with Se-donors are strong ones.

Considering the Δ*d*(I−Y) parameter, this classification can be extended to IBr and ICl adducts under the approximation that the range of Δ*d*(I−I) defining the three categories for I_2_ adducts are roughly valid also for IBr and ICl adducts: values of Δ*d*(I−Y) lower than 0.18 Å are indicative of weak or medium-weak adducts; Δ*d*(I−Y)> 0.34 Å is indicative of a very strong donor/acceptor interaction; 0.18 Å < Δ*d*(I−Y)< 0.34 Å corresponds to strong adducts. Figures 4 and 5 clearly show that IBr and ICl adducts with both S- and Se-donors are strong adducts [63, 65, 66, 68].

**Figure 6:**
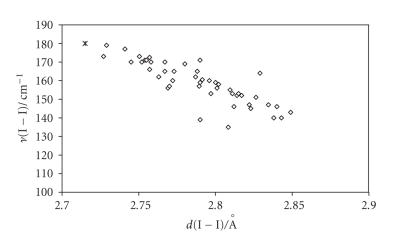
Scatter plot of *ν* (I−I)/cm^−1^ versus *d*(I−I)/ Å for weak or medium-weak adducts (⋄, data from [13, 14, 17–19, 21, 22, 25, 28, 30–33, 39, 43–46, 48, 54–56, 59, 60, 67]), solid diiodine (∗) [97].

**Figure 7:**
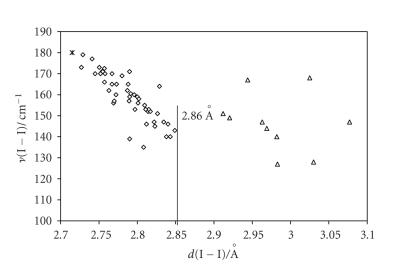
Scatter plot of *ν* (I−I)/cm^−1^ versus *d*(I−I)/ Å for weak or medium-weak adducts (⋄, data from [13, 14, 17–19, 21, 22, 25, 28, 30–33, 39, 43–46, 48, 54–56, 59, 60, 67]), solid diiodine (∗) [97], and strong adducts (Δ, data from [14, 44, 45, 48, 71, 72, 81, 97]).

**Figure 8:**
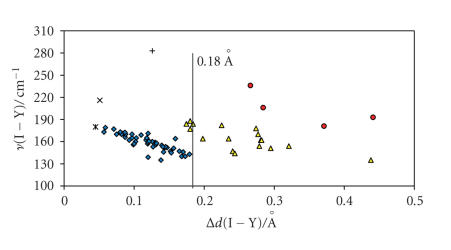
Scatter plot of *ν* (I−Y)/cm^−1^ versus Δ*d*(I−Y)/ Å [Y=I(⋄) [13, 14, 17–19, 21, 22, 25, 28, 30–33, 39, 43–46, 48, 54–56, 59, 60, 67], Br (Δ) [6, 59, 60, 62–67, 81], Cl(o) [60, 67, 68]]. ∗ = solid diiodine [97], × = solid IBr [98], + = solid ICl [99].

This classification was initially introduced to bring order among FT-Raman data recorded for a large number of structurally characterized I_2_ adducts [97]. Indeed for weak or medium-weak I_2_ adducts (*d*(I−I) < 2.86 Å) a linear correlation was found between the measured *ν*(I−I) Raman frequency and the I−I bond length, with *ν*(I−I) shifted towards lower values (in the range 180–135 cm^−1^) as compared to the stretching frequency of I_2_ at the solid state (180 cm^−1^) [97] as a consequence of adduct formation (Figure 6). For strong I_2_ adducts, two main peaks are generally detected in their FT-Raman spectra, ascribable to the symmetric (*ν*_1_, 120–115 cm^−1^) and antisymmetric (*ν*_3_, 145–125 cm^−1^) stretching modes of the E−I−I three-body system (E = S, Se); a much less intense peak in the range 100–80 cm^−1^ due to a bending mode (*ν*_2_) is also observed (lower attention will be paid to this vibrational mode in this paper) [65, 71, 81]. Figure 7 clearly points out the differences in terms of FT-Raman behavior between weak and strong I_2_ adducts; in fact, the antisymmetric (*ν*_3_) stretching frequency (having a major contribution from the I−I stretching) observed for the strong adducts does not fall within the linear correlation *ν*(I−I) versus *d*(I−I) found for weak I_2_ adducts. For IBr and ICl adducts, which are strong adducts according to the above classifications, much less structural and FT-Raman data are available, therefore generalizations are less reliable. IBr adducts with both S- and Se-donors show one main peak in their FT-Raman spectra in the range 190–140 cm^−1^ [16, 59, 60, 62–67, 81] at a lower frequency with respect to solid IBr [216 cm^−1^, *d*(I−Br) = 2.521(4) Å] [98], and it is assignable to a stretching vibration of the E−I−Br three-body system having a major contribution from the *ν*(I−Br) vibration [63]. ICl adducts (only four out of seven are both structurally and vibrationally characterized) [60, 67, 68] generally show in their FT-Raman spectra two main peaks: one in the range 240–180 cm^−1^ presumably due to the antisymmetric (*ν*_3_) stretching vibration of the E−I−Cl three body-system (E = S, Se), and the other at about 130 cm^−1^ due to the symmetric (*ν*_1_) stretching vibration (solid ICl is characterized by a single peak at 283 cm^−1^ in its FT-Raman spectrum with a *d*(I−Cl) = 2.446(6) Å) [99]. Interestingly, by considering the Δ*d*(I−Y) parameter (Y = I, Br, Cl), a linear correlation appears also to exist between Δ*d*(I−Br) and *ν*(I−Br) for IBr adducts, and between Δ*d*(I−Cl) and the *ν*(E−I−Cl) stretching mode corresponding to the *ν*_antisym_ in symmetric three-body systems, for ICl adducts (Figure 8).

Very few examples of very strong adducts with chalcogen donors are known, for which no vibrational characterization has been reported. Very strong adducts between group-15 donors (P, As, Sb) and dihalogens/interhalogens are more numerous [100–104]. The vibrational properties of these systems reflect the nature of the [D−X]^+^ cation interacting with a Y^−^ anion [100, 101].

The classification for the CT adducts based on the I−Y bond order can also be extended to trihalides such as XY_2_^−^ (X = I, Br; Y = I, Br, Cl). In fact, these can be formally considered CT adducts between a Y^−^ anionic Lewis base and an XY Lewis acid. Under this point of view, symmetrical or slightly asymmetrical trihalides can be considered belonging to the class of strong adducts, whereas strongly asymmetric trihalides can be considered belonging to the class of weak adducts. Spectroscopic implications of this are analyzed below. Usually a three-centre, four-electron (3c, 4e) bonding scheme is applied to these triatomic anions. This accounts for the 0.5 bond order calculated in symmetric systems (the empty *p*_z_ orbital of a 6-electron low-spin central X^+^ cation interacts, in the *D*_∞h_ point group, with the out-of-phase symmetry orbital combination (*σ*_u_^+^ in *D*_∞h_) obtained from the lone pairs of two terminal Y^−^ anions to produce a bonding and an antibonding MO, the other symmetry orbital combination (*σ*_g_^+^ in *D*_∞h_) becoming a nonbonding orbital) [65]. Some authors have extended this description to the three-body system E−X−Y in CT adducts between chalcogen donors and dihalogens/interhalogens [61, 65], thus pointing out the strict structural and spectroscopic analogy of these compounds with trihalides. Before considering these analogies more in detail, it is better to describe from a structural and spectroscopic point of view the class of compounds known as polyiodides which apparently have nothing to share with CT adducts of chalcogen donors with dihalogens and interhalogens.

## TRIIODIDES AND HIGHER POLYIODIDES SPECIES

It is well known that I_2_ is the dihalogen having the highest ability to catenate, thus affording oligomeric polyanions which can assume a wide range of structural motifs [105, 106]. This tendency to catenate decreases considerably on passing to dibromine and dichlorine [107].

Most of the known polyiodides have the general formula (I_2*m*+*n*_)^*n*−^ which formally implies the addition of *m* I_2_ molecules to *n* iodide ions. Examples of small polyiodides belonging to this family, such as I_3_^−^, I_4_^2−^, and I_5_^−^, are very numerous in literature, but the occurrence of discrete I_2_-rich higher polyiodides (from I_7_^−^ to I_22_^4−^) becomes increasingly rare as *m* and *n* increase [105, 106]. On the basis of structural data, all known higher discrete polyiodides can be considered derived from the donor/acceptor interaction of asymmetric I_3_^−^ and/or I^−^ with I_2_ molecules that emerge slightly elongated [I−I ∼ 2.75–2.80 Å, (I_3_^−^)I ^−^ ⋯ I_2_ ∼ 3.2–3.6 Å]. ∠ (I_3_^−^) I^−^−I−I bond angles are frequently observed at 90 or 180° but can deviate considerably from these values with longer (I_3_^−^)I ^−^ ⋯ I_2_ bond lengths. Polyiodides can be regarded, therefore, as weak or medium-weak adducts of the type [(I ^−^)_*n*−*y*_ · (I_3_^−^)_*y*_· (I_2_)_*m*−*y*_], whose geometrical and topological features can be very different and often unpredictable. Some of these polyiodides are present in the crystal lattice as discrete aggregates, but they frequently form polymeric chains or extended 2D or 3D networks in the polyanionic matrix via I ⋯ I cross-linking soft-soft secondary interactions: these generally range from 3.6 Å up to the van der Waals sum for two iodine atoms (4.3 Å), and the identification of the basic polyiodide unit can became arbitrary. This extraordinary ability of I_2_, I_3_^−^, and I^−^ to interact with each other to give polyiodides is affected profoundly by the size, shape, and charge of the associated countercation, and these parameters have been considered in recent papers to achieve control over their 3D architecture [92, 105, 106, 108–110].

From the above, it is clear that in the absence of a crystal structure determination, it becomes very hard to guess the nature and the structural features of polyiodide anions. The FT-Raman spectroscopy can only give valuable information on the nature of their building blocks.

In the linear and symmetric I_3_^−^, the Raman-active symmetric stretch (*ν*_1_) occurs near 110 cm^−1^, while the antisymmetric stretch (*ν*_3_) and the bending deformation (*ν*_2_) are only infrared-active. The latter two modes become Raman-active for asymmetric I_3_^−^, in which case they are found near 134 (*ν*_3_) and 80 cm^−1^ (*ν*_2_), having medium and medium-weak intensities, respectively, as found for strong CT I_2_ adducts. For highly asymmetric I_3_^−^ ions, which can be considered weak adducts between I^−^ and I_2_ [I ^−^·I_2_], as found in neutral I_2_ adducts with S-donors, the FT-Raman spectrum shows only one strong band in the range 180–140 cm^−1^, indicative of the presence of a perturbed I_2_ molecule [106, 111, 112].

As already mentioned, all the higher polyiodide species may be regarded as weak or medium-weak adducts of the type [(I^−^)_*n*−*y*_· (I_3_^−^)_*y*_· (I_2_)_*m*−*y*_]. Consequently, the corresponding FT-Raman spectra will show peaks due to perturbed diiodine molecules for [(I^−^)_*n*_· (I_2_)_*m*_] systems (*y* = 0), and characteristic peaks due to both perturbed diiodine molecules and symmetric or slightly asymmetric I_3_^−^ ions for polyiodies of the types [(I_3_^−^)_*n*_ · (I_2_)_*m*−*n*_] (*n* = *y* ≠ 0) and [(I^−^)_*n*−*y*_ · (I_3_^−^)_*y*_ · (I_2_)_*m*−*y*_] (*n* > *y* ≠ 0). It is therefore evident that except for the presence or absence of symmetric and slightly asymmetric I_3_^−^ units, the Raman technique is unable to distinguish between the different types of polyiodides or to discriminate unambiguously between the polyiodides and the neutral I_2_ adducts with chalcogen donors. However, it can give valuable information on the extent of the lengthening of the I−I bond, whether or not it has been produced by interaction of I_2_ with a neutral donor or an ion. Furthermore, FT-Raman spectroscopy cannot give any structural information on the topological features of an extended polyiodide network as the technique cannot elucidate the structure beyond the basic polyiodide units in terms of combinations of I^−^, I_2_, and I_3_^−^ units.

A further complication to the interpretation of FT-Raman spectra of polyiodides may arise when the basic polyiodide unit sits on special crystallographic positions. For example, in [Ag([18]aneS_6_)]I_7_ [113], the complex cation is embedded in a 3D polymeric polyiodide matrix of I_7_^−^ anions. The overall structure of the [(I_7_)^−^]_∞_ network can best be described as a distorted cube in which I^−^ ions occupy the lattice points of a primitive rhombohedral lattice with one slightly elongated I_2_ molecule placed along each edge bridging two I^−^ ions. Each I^−^ interacts with six diiodine molecules arranged in a perfect *D*_3d_ symmetry. Because all six I_2_ molecules have the same I−I bond distance, only one peak should be present in the FT-Raman spectrum below 180 cm^−1^. However, the stretching vibrations of the six individual I_2_ units can combine, and in *D*_3d_ symmetry they give rise to two Raman-active normal modes of A_1g_ + E_g_ types. The observed bands at 179 and 165 cm^−1^ can therefore be assigned to these two modes, respectively. A lowering of the symmetry due to different bond distances for the two perturbed I_2_ units will split the E_g_ mode, thus causing the appearance of three bands in the FT-Raman spectrum. Similarly, the case of the I_5_^−^ ion with a C_2v_ symmetry in [Ag([9]aneS_3_)_2_]I_5_ [113] can be tackled: the vibrations of the two individual I_2_ units combine to give normal modes of the A_1_ + B_2_ types. A lowering of the symmetry due to different bond distances for the two perturbed I_2_ units will increase the energy of the higher energetic stretching normal mode and lower the energy of the lower energetic stretching normal mode.

It may also happen that polyiodides are unstable under the laser beam and cause spurious peaks to appear in their Raman spectra. This is more likely using visible excitation sources and resonance Raman spectroscopy; using near-infrared laser excitation sources and FT-Raman spectroscopy, such problems, particularly fluorescence and photoreactions, can be considerably reduced. Nevertheless, decomposition of polyiodides during spectrum acquisition must be always considered and ascertained before passing on to the assignment of the FT-Raman bands in order to avoid confusion with the scattering from decomposition products (generally driven from loss of diiodine molecules).

After this concise overview on polyiodides, it is worthy to point out the vibrational analogies in terms of FT-Raman that can exist between I_3_^−^ and I_2_ adducts with chalcogen donors.

Strong CT I_2_ adducts, in particular those formed by Se-donors, present two main peaks in their FT-Raman spectra assigned to the antisymmetric and symmetric stretching modes of the Se−I−I three-body system (see above). The observed frequencies are very close to those normally recorded for asymmetric triiodides. On the other hand, weak or medium-weak CT I_2_ adducts, in particular those with S-donors, present only one peak in their FT-Raman spectra assigned to the stretching mode of the perturbed diiodine molecule (see above). The observed frequency is indistinguishable from that recorded for very asymmetric triiodides. Thus the groups Se−I−I and (I−I−I)^−^, and S ⋯ I−I and I ^−^ ⋯ I−I give very similar FT-Raman spectra. This fact can produce confusion when chalcogen donors are reacted with diiodine, and no X-ray diffraction analysis of the products is available: the formation of a triiodide, and, more broadly of a polyiodide, can be erroneously invoked in the presence of neutral adducts and vice versa.

## HYPERVALENT CHALCOGEN COMPOUNDS

The pivotal role of the vibrational properties of I_3_^−^ and other trihalides in the assignment of the FT-Raman peaks for the products obtained by reacting chalcogen donors with dihalogens/interhalogens is even clearer by considering the class of hypervalent compounds.

**Figure 9:**
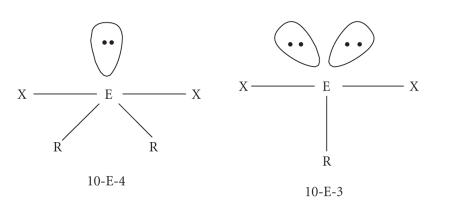
Schematic representation of 10-E-4 and 10-E-3 hypervalent chalcogen compounds (E = S, Se, Te).

Hypervalent chalcogen compounds featuring a linear X−E−Y moiety [X = Y = I, Br, Cl; X = I, Y = Br, Cl; E = S, Se] can be considered to derive formally from the oxidative addition of an X_2_ or XY molecule to the donor molecule containing the chalcogen atom. With donors of the types R_2_C=E (E = S, Se) and R_2_E (E = S, Se), the structural features of the corresponding adducts is well explained by the VSEPR model, according to which the geometry at the chalcogen atom is a pseudotrigonal bipyramid (tbp) with the halogens occupying the apical positions, in the case of R_2_C=E donors (two lone pairs and one bond pair in the plane perpendicular to the X−E−Y direction), and disphenoidal in the case of R_2_E ones (one lone pair and two bond pairs in the plane perpendicular to the X−E−Y direction). These compounds are commonly referred to as, respectively, 10-E-4 and 10-E-3 systems, indicating that the chalcogen atom E is formally associated with five pairs of electrons, only four or three of which are bond pairs (Figure 9), respectively [114]. As with a trihalide or a CT adduct (see above), the chemical bond in the X−E−Y fragment can be described using the 3c, 4e bonding scheme, which implies a total bond order of 1 (0.5-bond order for each E−X bond in symmetric systems). This description agrees with the qualitative observation that on increasing the electronegativity difference between the halogen and the chalcogen, hypervalent chalcogen adducts are formed more easily than CT adducts bearing an E−X−Y linear group on reacting chalcogen donors with dihalogens and interhalogens. Indeed, no hypervalent sulfur compounds containing the I−S−I moiety are known, and only three hypervalent selenium compounds containing the I−Se−I framework have recently been structurally characterized [71, 115]. Only three examples of a Br−S−Br type hypervalent sulfur compound with dibromine have been structurally characterized [59, 88, 116], while analogous compounds from selenium containing substrates are numerous [65, 69, 85, 88, 117]. As expected, hypervalent sulfur and selenium compounds containing the linear Cl−E−Cl (E = S, Se) group are very well known [65, 69, 86]. For the oxidative addition of interhalogens (IBr, ICl), only two examples of “T-shaped” adducts featuring I−E−Br (E = S, Se) moieties are known (for the hypervalent compound featuring the I−S−Br fragment, no X-ray characterization is reported) [89, 116].

The strict analogy between trihalides and hypervalent chalcogen compounds is clearly pointed out also by the Raman spectroscopy. In fact, it has been shown that hypervalent Se-compounds featuring a linear I−Se−I moiety show in the low-frequency region of their FT-Raman spectra one or two peaks depending on whether the I−Se−I fragment is symmetric or slightly asymmetric, which are very similar to those arising from a symmetric or asymmetric I_3_ ^−^ [71]. Therefore, the groups Se−I−I (strong adducts), (I−I−I)^−^ (triiodides), and I−Se−I (hypervalent compounds) can be undistinguishable from a Raman point of view.

**Figure 10:**
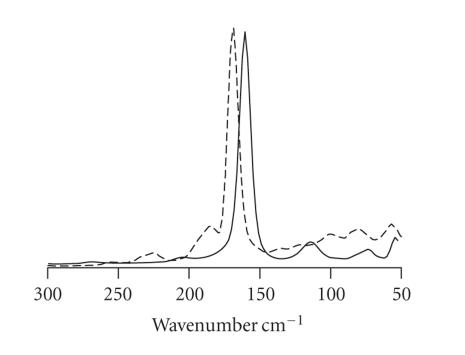
Superimposed FT-Raman spectra of the symmetric Se-hypervalent adduct *N,N′*-dimethylbenzimidazole-2(3H)-selone·Br_2_ (full line) and the salt (HL′)^+^ Br_3_^−^ (2, 4, 6-tris(2-pyridyl)-1, 3, 5-triazinium tribromide, dashed line) featuring a symmetric Br_3_^−^ [118].

**Figure 11:**
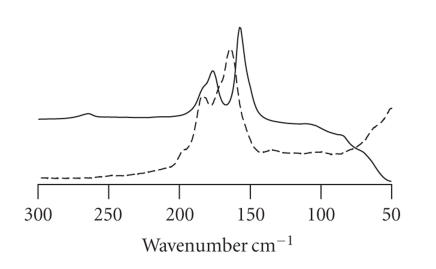
Superimposed FT-Raman spectra of the asymmetric Se-hypervalent adduct *N,N′*-dimethylimidazolidine-2-selone·Br_2_ (full line) and the salt (H_2_L′)^2+^ Br^−^IBr_2_^−^ (2, 2′-dipyridinium disulfide bromide iododibromide, dashed line) featuring an asymmetric IBr_2_^−^ [118].

The same analogy is also found for hypervalent chalcogen compounds featuring a Br−E−Br linear system (E = S, Se). In fact, the vibrational properties of a Br−E−Br group resemble those of (Br−X−Br)^−^ anions (X = I, Br) [65, 66, 88, 117]. The FT-Raman spectrum of a symmetrical Br−E−Br group only shows one Raman peak near 160 cm^−1^ (see Figure 10), as found in symmetric Br_3_^−^ and IBr_2_^−^ anions, which can be assigned to the symmetric stretching vibration of the three-body system. Asymmetric Br−E−Br groups display an additional and generally less intense peak at around 190 cm^−1^ (see Figure 11), as found for asymmetric Br_3_^−^ and IBr_2_^−^ anions, which is assigned to the antisymmetric stretching vibration of the Br−E−Br or (Br−X−Br)^−^ three-body systems (E = S, Se; X = I, Br). These analogies are quite evident from Figures 10 and 11 [118]. Unfortunately, in the literature no spectroscopic data are available for chalcogen-hypervalent Cl_2_ adducts, thus preventing a structural/vibrational comparison of the Cl−E−Cl (E = S, Se) framework with the anions (Cl−X−Cl)^−^ (X = I, Br). Overall, we can say that strong I_2_ adducts (generally deriving from Se-donors), XY_2_^−^ trihalides (X = I, Br; Y = I, Br, Cl), and hypervalent chalcogen compounds featuring a linear X−E−X moiety (X = I, Br, Cl; E = S, Se) can all be described with the same MO bonding scheme (3c, 4e) and show very similar vibrational properties whose features depend on whether they are symmetric or asymmetric. On the other hand, weak I_2_ adducts (generally feturing S-donors) have FT-Raman spectra similar to those recorded for very asymmetric triodides or polyiodides of the type [(I ^−^)_*n*_ · (I_2_)_*m*_].

## TWO CHALCOGEN-COORDINATED HALOGEN(I) COMPLEXES

Salts of two-chalcogen-coordinated halogen(I) complexes [(LE−X−EL)^+^] can be formally considered as a central X^+^ (X = I, Br, Cl) coordinated by two donor molecules. The chemical bond in the resulting E−X−E almost-linear framework can be described according to 3c, 4e bonding scheme, as for CT adducts, trihalides, and hypervalent chalcogen compounds. So far, only cations of this kind formally featuring a central I^+^ interacting with either S- or Se-donors have been isolated from the direct reaction of chalcogen donors and dihalogens (see Devillanova et al in this issue of BC&A), and have been structurally characterized [43, 45, 65]. Similarly to what is observed for the three-body system in CT adducts (E−I−Y, E = S, Se; Y = I, Br, Cl), trihalides (X−I−X, X = I, Br, Cl), and hypervalent compounds (X−E−X, E = S, X = Br, Cl; E = Se, X = I, Br, Cl), also in these cations there is a correlation between the two E−I bond distances (E = S, Se): the reinforcement of one I−E bond corresponds to a lengthening of the other, the total length of the E−I−E framework being almost independent of the nature of the substrate incorporating the chalcogen. The mean value of the E ⋯ E distance is 5.28 Å for S−I−S and 5.50 Å for Se−I−Se systems (these distances are very similar, resp, to the averaging value for the sums of S−I and I−Cl in ICl adducts with S-donors (5.22 Å), and Se−I and I−Br in IBr adducts with Se-donors (5.53 Å)).

Unfortunately, very few spectroscopic data are available for iodonium salts in the literature, and generally the FT-Raman spectra are dominated by the absorption peaks due to the polyiodide counteranions. Therefore, a structural/vibrational relationship cannot be established. However, on the grounds of what has been said, and considering S/Cl and Se/Br mass similarities, the Raman peaks for the stretching vibrations of the E−I−E (E = S, Se) three-body systems could fall, depending on the organic framework, at frequencies reasonably close to those observed for ICl adducts with S-donors or ICl_2_^−^ trihalides (E = S), and IBr adducts with Se-donors or IBr_2_^−^ trihalides (E = Se).

## CONCLUSIONS

The results reviewed in this paper clearly point out that the reactions of chalcogen donors with dihalogens or interhalogens can afford a great variety of products depending on the nature of the donor, the reaction molar ratio, and the experimental conditions (solvent and temperature). In the absence of an X-ray diffraction analysis, the FT-Raman spectroscopy can be of help in elucidating the nature of the products obtained. However, much attention must be paid in the assignment of the Raman peaks recorded in order not to make confusion. In fact, the vibrational behavior in the low-frequency region is sometimes undistinguishable for very similar three-body systems: E−I−Y (E = S, Se; Y = I, Br, Cl) in CT adducts, X−E−X (E = S, X = Br, Cl; E = Se, X = I, Br, Cl) in hypervalent chalcogen compounds, and E−I−E (E = S, Se) in two chalcogen-coordinated halogen(I) complexes, which can all be described according to a 3c, 4e bonding scheme. Very recently, a vibrational analogy has also been found between I_2_ adducts of Se-donors and complexes of bidentate phosphate selenide ligands with mesitylenetellurenyl iodide featuring a Se−Te−I linear systems [119]. The problem is even more complex if the vibrational analogy with trihalides IY_2_^−^ (Y = I, Br, Cl) is considered. For example, the groups Se−I−I (strong adducts), I_3_^−^ (asymmetric triiodides), and I−Se−I (hypervalent compounds) are undistinguishable from a Raman point of view, as well as the Br−E−Br group (E = S, Se) being vibrationally very similar to Br_3_^−^ and IBr_2_^−^ anions.
